# Voltage-Controlled Skyrmionic Interconnect with Multiple Magnetic Information Carriers

**DOI:** 10.1021/acsami.2c07470

**Published:** 2022-06-25

**Authors:** Runze Chen, Yu Li

**Affiliations:** † Department of Computer Science, School of Engineering, The University of Manchester, Manchester M13 9PL, United Kingdom; ‡ Frontier Institute of Chip and System, Fudan University, Shanghai 200433, China

**Keywords:** magnetic skyrmions, spintronic devices, magnetic anisotropy, interconnect device, micromagnetic simulations, VCMA, skyrmionic spin textures

## Abstract

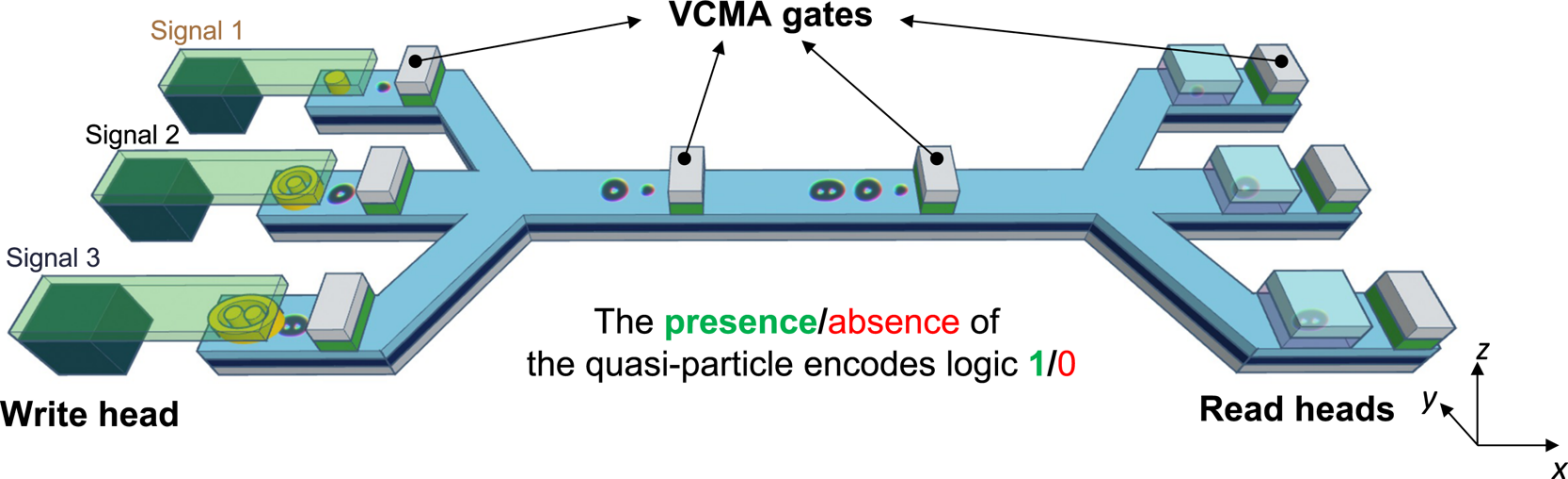

Magnetic skyrmions have been in the spotlight since they were observed in technologically relevant systems at room temperature. More recently, there has been increasing interest in additional quasiparticles that may exist as stable/metastable spin textures in magnets, such as the skyrmionium and the antiskyrmionite (i.e., a skyrmion bag with two skyrmions inside) that have distinct topological characteristics. The next challenge and opportunity, at the same time, is to investigate the use of multiple magnetic quasiparticles as information carriers in a single device for next-generation nanocomputing. In this paper, we propose a spintronic interconnect device where multiple sequences of information signals are encoded and transmitted simultaneously by skyrmions, skyrmioniums, and antiskyrmionites. The proposed spintronic interconnect device can be pipelined via voltage-controlled magnetic anisotropy (VCMA) gated synchronizers that behave as intermediate registers. We demonstrate theoretically that the interconnect throughput and transmission energy can be effectively tuned by the VCMA gate voltage and appropriate electric current pulses. By carefully adjusting the device structure characteristics, our spintronic interconnect device exhibits comparable energy efficiency with copper interconnects in mainstream CMOS technologies. This study provides fresh insight into the possibilities of skyrmionic devices in future spintronic applications.

## Introduction

Magnetic skyrmions are nontrivial particle-like spin textures stabilized in noncentrosymmetric bulk magnets and thin magnetic films with broken inversion symmetry. This behavior is made possible by the interactions favoring nonparallel magnetization orientation, such as the Dzyaloshinskii–Moriya interaction (DMI) and dipolar coupling.^1−3^ Skyrmionic spin textures can be characterized by topological indices, such as the skyrmion number, which counts how many times the vector field configuration wraps around a unit sphere and reflects the topological charge they may be endowed with. The skyrmion number is defined as
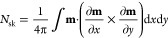
1where *N*_sk_ = ±1 accounts for the case of magnetic skyrmions, and the sign reflects their polarity. The skyrmionium, i.e., a skyrmion bag with one skyrmion with the opposite *N*_sk_ inside, is a magnetic quasiparticle with vanishing topology *N*_sk_ = 0 that has been proposed as advantageous for racetrack memory applications.^4^ More recently, several studies suggest that nanomagnets can host a plethora of topological quasiparticles, including both theoretical calculations^5,6^ and experimental demonstrations such as skyrmion bags in liquid crystals^7^ and skyrmion bundles^8^ as well as skyrmion clusters^9^ in chiral magnets. Such topological spin textures with different *N*_sk_ exhibit different dynamics behaviors under current-induced spin torques,^6,8^ which provides new ideas and directions for designing new skyrmionic functional devices.

On the basis of these results, the skyrmionic quasiparticles can be promising candidates for future low-power and low-temperature computing because of their nonvolatility, nanoscale size, and ease of manipulation.^10^ Field-free creation of skyrmionic quasiparticles is one of the key prerequisites to utilize magnetic skyrmions in realistic devices, which has been explored both theoretically^2,4,11^ and experimentally^12−18^ in recent years. To date, the use of skyrmionic quasiparticles has been proposed in both conventional computing and emerging computational paradigms, such as skyrmionic transistors,^19^ skyrmionic logic gates,^20−22^ skyrmionic racetrack memory,^9,23−25^ spintronic nano-oscillators,^26^ skyrmionic resonant diodes,^27^ skyrmion neuromorphic computing,^28,29^ and reservoir computing.^30^ However, apart from information processing, the efficient information transmission with spintronic devices, e.g., spintronic interconnect devices and multiplexers, has not received the required attention. Considering that interconnect energy and latency is often the bottleneck of modern computing systems, novel and effective interconnect is an indispensable requirement for the adoption of any emerging technology.

In complementary metal-oxide-semiconductor (CMOS) integrated circuits, interconnects link two or more circuit elements (i.e., transistors) electrically,^31^ where copper wires are commonly utilized. However, the power spent on copper interconnects can exceed the energy spent for computation, which becomes a critical challenge toward delivering exascale performance at a reasonable power budget.^31^ It is therefore desirable to address the problem by exploiting emerging technologies. Optical interconnects are promising energy-efficient solutions for long-distance and parallel data transmission.^32^ Meanwhile, silicon photonic interconnects with the ability to use CMOS-compatible fabrication have made significant strides that can benefit many applications, e.g., data centers, high-performance computing, and sensing.^33^ Here, we explore alternative nonvolatile and energy efficient information transfer through signal multiplexing using spintronic devices. In our recent work, a prototype of notch-based interconnect device was proposed,^34^ which can perform topological filtering that enables signal multiplexing utilizing sequences of magnetic skyrmions and skyrmioniums. The notch-based nanotrack design has been frequently utilized in recent numerical studies.^11,20,34^ However, to avoid insidious risks that include likelihoods of pinning and annihilation at notches/edges and to achieve effective performance tunability require the design of more realistic and robust spintronic interconnect devices.

We therefore propose a spintronic interconnect device in a ferromagnetic nanotrack with voltage-controlled magnetic anisotropy (VCMA) gates, and we deliver a systematic study of its operation and performance via theoretical calculations and micromagnetic simulations. The proposed interconnect device utilizes multiple magnetic quasiparticles as information carriers, i.e., the topologically nontrivial spin textures skyrmions, skyrmioniums, and antiskyrmionites (i.e., the skyrmion bag with two skyrmions inside, exhibiting the opposite topological charge to a magnetic skyrmion). It is worth mentioning that the notation of the antiskyrmionite in this work is for consistency to our previous publication.^34^ This approach exhibits superior thermodynamic stability than notch-based devices, as evidenced by our thermal stability analyses on the nanotrack. We then demonstrate the effective tunability of the device performance and information transmission energy. Finally, the proposed pipelined spintronic interconnect, achieved via VCMA gates, shows a comparable energy efficiency with copper interconnects in CMOS by carefully optimizing the nanotrack geometry. Our proposal may provide inspiration for not only material scientists but also device engineers in utilizing multiple skyrmionic quasiparticles to build energy-efficient nanodevices in future spintronic nanocomputing applications.

## Results

### VCMA-Based Spintronic Interconnect Device

**Figure 1:**
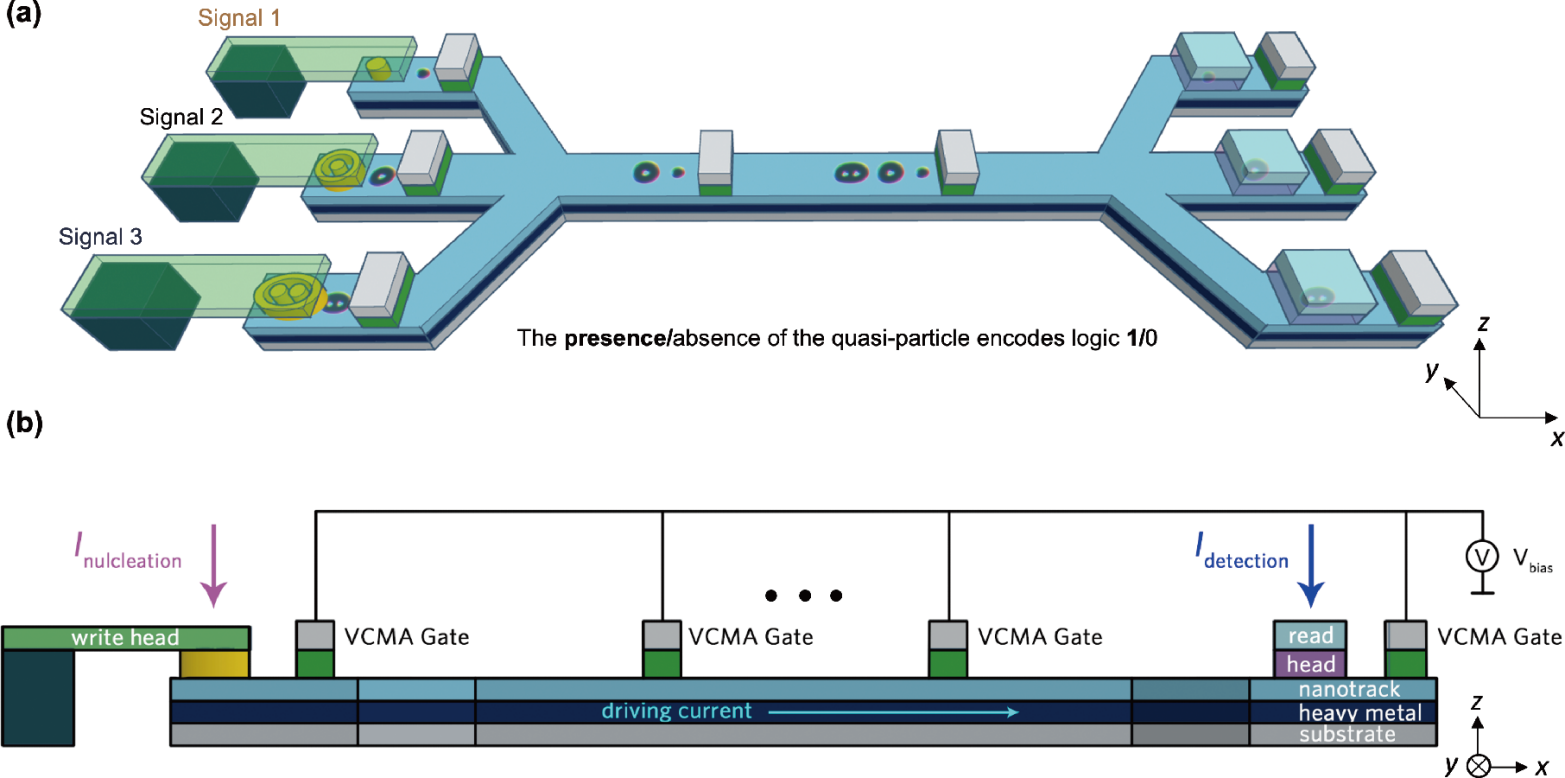
Schematic drawing of the proposed voltage-controlled spintronic interconnect device: (a) perspective view of the device; (b) cross-sectional view. The proposed device comprises spin-transfer torque (STT) write heads, VCMA-controlled gates, nanotrack, and magnetic tunnelling junction (MTJ)-based read heads.

The proposed spintronic interconnect device is schematically depicted in Figure 1. The spintronic interconnect consists of four key modules: (1) the three write heads in the left of Figure 1a, which nucleate the corresponding magnetic quasiparticles according to the signal from the interface circuits, as a three-branch encoder; (2) the ferromagnetic (FM)/heavy metal (HM) heterostructure nanotrack supporting the propagation of the carrier streams via the spin-orbit torque (SOT); (3) VCMA-based gates distributed evenly on the nanotrack serving as synchronizers; (4) the three read heads detecting the presence/absence of a quasiparticle in the corresponding branch.

As shown in Figure 1, there is a three-branch multiplexer on the left side and a three-branch demultiplexer on the right side. Signals are encoded (nucleated) through the three-branch multiplexer into the nanotrack and decoded (detected) via the three-branch demultiplexer. As shown in Figure 1a, the magnetic quasiparticles can be nucleated by the write heads via perpendicularly injected electric current pulses and current-induced spin-transfer torques. The electrical nucleation method of the magnetic quasiparticles has been discussed in detail and simulated in refs (4, 11, and 35) as well as our previous work.^34^ The SOT achieves the transmission, and the intrinsic skyrmion Hall effect (SkHE) enables an automatic topological filtering process. The device can work bidirectionally because of the symmetry of this design and the propagation properties of skyrmionic quasiparticles; our interconnect device may also be relevant to reversible computing.^36^ Because the quasiparticles present slightly different velocities under SOT on the nanotrack,^6^ it is crucial to introduce synchronizers in the device both for precise detection and information integrity.^34^ It should be noted that the magnetic tunnelling junction (MTJ) reading heads are illustrated for signal detection in Figure 1 merely as an example. Besides MTJ detectors,^37^ we could also utilize other detection techniques, such as measuring the topological Hall effect (THE)^4,38^ and hinge spin polarization.^39^ Note that the hinge spin polarization was first proposed in magnetic topological insulators, but the feature is general and, therefore, relevant to the ferromagnetic and the antiferromagnetic phases as a potential detection method.

The perpendicular magnetic anisotropy (PMA), also called perpendicular uniaxial anisotropy, can be locally modulated by applying a voltage in thin films,^19,23,40−45^ which is known as the voltage-controlled magnetic anisotropy (VCMA) effect and provides great potential in practical applications. The VCMA effect was first reported in 3d-transition ferromagnetic materials in 2007,^45^ where a coercivity change was observed in 2–4 nm thick FePt and FePd films immersed in a liquid electrolyte. Opposite trends in the change in coercivity depending on the applied voltage were also observed. Surprisingly, Maruyama et al. reported that an electric field of about 100 mV nm^–1^ can change PMA of a bcc Fe(001)/MgO(001) junction by 40% with the VCMA efficiency ϑ of 210 fJ V^–1^ m^–1^ at room temperature.^44^ The VCMA efficiency ϑ is defined as the ratio between the voltage-induced change of total uniaxial anisotropy near the interface (in units of J m^–2^) and the applied electric field (in units of V m^–1^).^46^ The micromagnetic simulation of the VCMA effect in this work is based on a linear relationship describing the effective contribution of the applied electric field to the uniaxial magnetocrystalline anisotropy constant:^23,42−44^
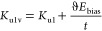
2where *E*_bias_ is the applied bias electric field on the VCMA gate (in units of V m^–1^), *K*_u1v_ is the resulting anisotropy constant after the electric field application, *K*_u1_ is the initial uniaxial anisotropy constant (in units of J m^–3^), and *t* is the thickness of the FM layer. The region with higher PMA can provide an extra barrier, whereas the one with lower PMA offers a potential well. In the remaining of this paper, we refer to the region with higher/lower PMA as a VCMA barrier/well. It should be noted that the material underneath VCMA gates can be preset with higher PMA values, which can be achieved by locally modulating material properties during the deposition process.^22^ As a result, the quasiparticles will be stopped by the barrier without voltage supply and move through the voltage-gated region with reduced PMA by applying negative bias voltage *V*_b_. Therefore, no voltage supply of VCMA gates is required between clock cycles in this design, significantly reducing the amount of leaked charge and static power dissipation.

In this work, magnetic quasiparticles are simulated in a single FM/HM bilayer heterostructure without thermal effects. We have already demonstrated that, by introducing a tailored magnetic multilayer structure interconnect, skyrmionic quasiparticles under finite temperature can exhibit stable behaviors similar to that in thermal-free systems.^28,34^ Therefore, simulations on the thermal fluctuation at finite temperature are not considered in the main results of this paper. Micromagnetic simulations are performed using the open-source package MuMax3.^47^ Calculations of equilibrium states of topological spin textures and minimum energy paths (MEPs) are performed using Fidimag,^48^ where the nudged elastic band method (NEBM) method is used for the calculation of MEPs between the equilibrium states. With this method, the energy barriers within transitions can be quantified. Magnetic parameters and the detailed configurations utilized in the simulations are introduced in Methods.

### Topological Properties of the Magnetic Quasiparticles

When a skyrmion moves along the nanotrack driven by the electric current, the Magnus force stemming from the spin precession causes its movement along a trajectory at an angle to the direction of the applied current, which is the well-known skyrmion Hall effect (SkHE).^49,50^ As for other types of magnetic quasiparticles, a similar deflection angle related to their topological charge can also be obtained.^6,8^ The SkHE is usually deemed harmful to skyrmionic devices because it affects the device performance and robustness, leading to the annihilation of skyrmions at the boundaries.^51^ Several strategies are thus proposed to suppress the SkHE, such as stabilizing skyrmions in ferrimagnetic material systems^52−55^ and synthetic antiferromagnets (SAF) structures,^56^ adding high-*K* materials at boundaries,^20,27^ modifying the racetrack structure,^57^ and moderating the spin Hall angle.^58^ However, in this work, we take a different approach to exploit the SkHE rather than circumvent it, to enable device implementation with multiple information carriers. The proposed spintronic interconnect can conduct automatic demultiplexing at the decoder (three-branch structure shown on the right side of Figure 1a), which exploits topological filtering arising inherently from the SkHE. In this way we realize the combination of salient topological properties of magnetic quasiparticles with their practical applications.

**Figure 2:**
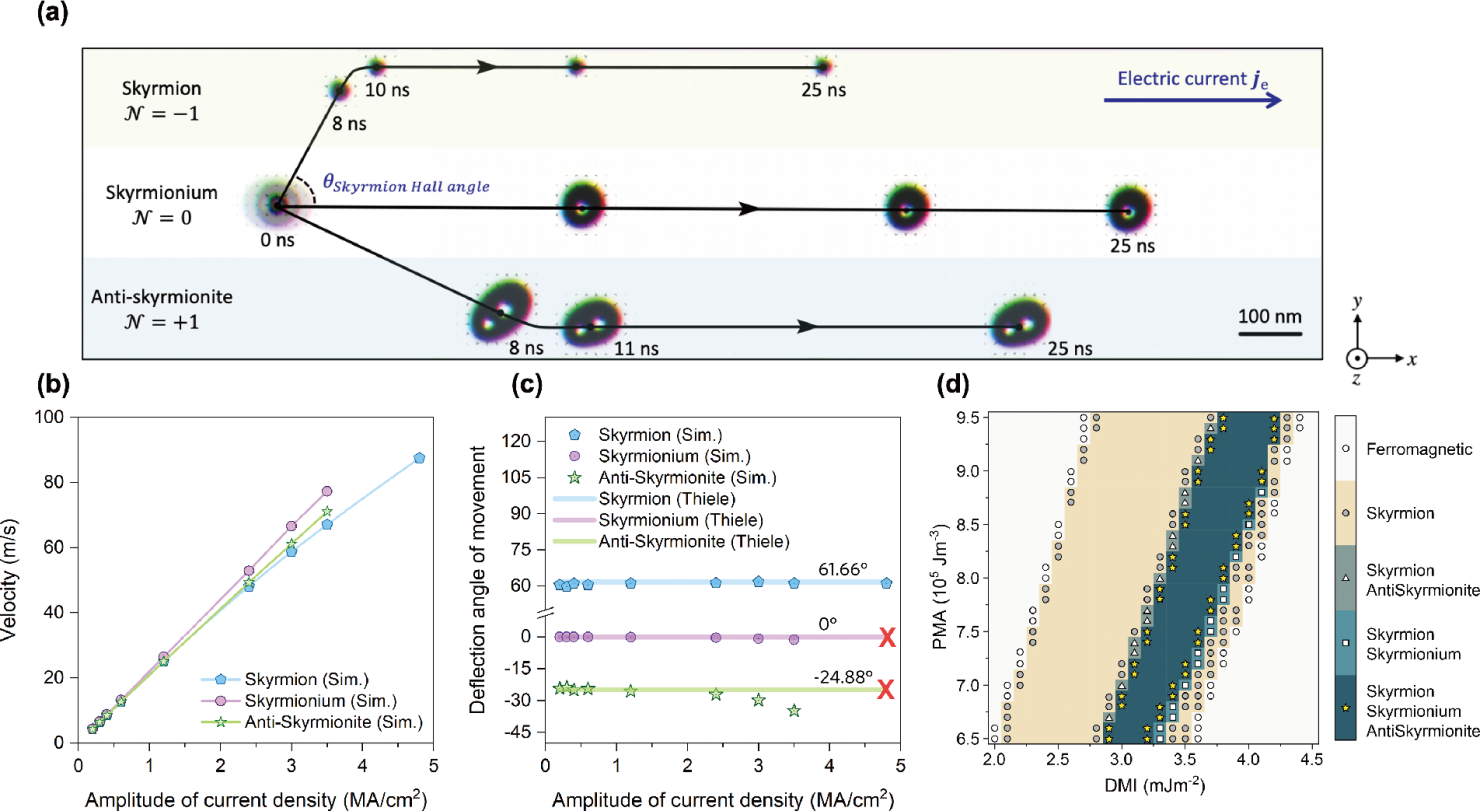
Skyrmionic quasiparticles Hall effect. (a) Micromagnetic simulation results of the propagation of a Néel skyrmion (*N*_sk_ = −1), a skyrmionium (*N*_sk_ = 0), and an antiskyrmionite (*N*_sk_ = +1) driven by a spin current perpendicularly injected to the *x*–*y* plane. (b) Velocities of quasiparticles propagating along the edge of the nanotrack (*x*-direction) as a function of the current density derived by simulations. (c) Hall angle of movement of the quasiparticles with respect to current densities both for simulations and theoretical calculations. The red crosses indicate that the skyrmionium and the antiskyrmionite annihilated when current densities >3.5 MA cm^–2^. (d) Magnetic stability phase diagram of the magnetic quasiparticles. The skyrmion, skyrmionium, and antiskyrmionite can coexist in magnetic systems with parameters colored in navy (the darkest color in the figure) and marked with yellow stars.

We performed micromagnetic simulations of a skyrmion, skyrmionium, and antiskyrmionite in a nanotrack under SOT. Electric current is applied in the HM layer underneath the FM layer such that a spin current will be injected perpendicular to the FM plane with the spin polarization in + *y* direction due to the spin Hall effect. Note that the skyrmion in our simulations carries a topological charge *N*_sk_ = −1 reflected by the negative polarity of the skyrmion core (spin ↓), whereas the antiskyrmionite delivers a total net topological charge of *N*_sk_ = +1, which acts as an effective antiskyrmion in the system. As shown in Figure 2a, the skyrmion (*N*_sk_ = −1) propagates first toward the +*y* direction and then along the direction of the driving current (+*x*); the antiskyrmionite (*N*_sk_ = +1) propagates first toward the -*y* direction and then along the direction of the driving current (+*x*). In contrast, the skyrmionium propagates strictly along the +*x* direction without any deflection. The skyrmion Hall angle θ_SkHE_ is defined as the angle between the trajectory of quasiparticles and the direction of applied current (+*x*), with positive sign when it is anticlockwise. From the simulation results, the skyrmion exhibits a larger absolute θ_SkHE_ than the antiskyrmionite, even though they share the same absolute topological charge. To highlight the reason for this difference, we then perform a theoretical analysis on the movement of quasiparticles under electrical current.

Micromagnetic simulations are performed by solving the Landau–Lifshitz–Gilbert (LLG) equation (see Methods), a time-dependent partial differential equation consisting of several terms. It is desirable to find an algebraic, practical description for the motion of noncollinear spin textures to better comprehend the results of spin dynamics simulations. By considering a magnetic quasiparticle as a rigid particle whose shape does not change significantly during the movement, the translational motion driven by the SOT can be described by a modified Thiele equation,^6,59,60^ which is an analytically solvable system of algebraic equations describing the velocity of magnetic quasiparticles
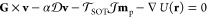
3where **G** = (0, 0, –4π*N*_sk_) is the gyroscopic vector with the topological charge *N*_sk_ defined in eq 1. **v** = (*v*_*x*_, *v*_*y*_) is the drifting velocity of a skyrmionic quasiparticle within the *x–y* plane. The first term **G** × **v** in eq 3 is the Magnus force that results in the transverse motion of skyrmions related to the spin precession, which directly results in SkHE.^49^ α is the dimensionless Gilbert damping parameter.  is the dissipative tensor and can be calculated by , where *M*_s_ is saturation magnetization. The term  quantifies the effect of the SOT driving the magnetic quasiparticle.  is the amplitude of SOT over the quasiparticle, where γ_e_ is the gyromagnetic ratio of an electron, ℏ is the reduced Planck constant,  is the electron current density, θ_SH_ is the spin Hall ratio (the ratio between the spin current and the electron current), *e* is the electron charge, and *t* is the thickness of the FM layer.  is the driving torque tensor and can be calculated by . **m**_p_ is the polarization orientation of the spin current due to the spin Hall effect. The fourth term ∇*U*(**r**) in eq 3 is an extrinsic force accounting for the interaction of a magnetic quasiparticle with other noncollinearities or the nanotrack edge.

As for the initial part of the motion after applying the SOT, we can obtain the skyrmion Hall angle of the quasiparticle as (for details, see Methods and ref (6))
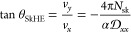
4After the transverse movement of the quasiparticle, supposing a significantly small current density, the quasiparticle moves along the direction of the driving current along the edge of the nanotrack, i.e., *v*_*y*_ = 0 and *v*_*x*_ ≠ 0, as demonstrated in Figure 2a. In this condition, the velocity of the quasiparticle *v*_*x*_ can be extracted as (for details, see Methods and ref (6))
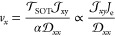
5To verify the theoretical predictions from eqs 4 and 5 derived from the Thiele equation, we performed micromagnetic simulations of an individual skyrmion, skyrmionium, and antiskyrmionite, respectively, to obtain their velocity *v*_*x*_ and skyrmion Hall angle θ_SkHE_ under increasing amplitude of current densities up to 5 MA cm^–2^. It should be noted that the similar order of magnitude of current densities have been utilized and reported in experiments.^9,29,49,61^ We thus believe the amplitude of current densities used in our work is safe on real thin-film-based devices.

As shown in Figure 2b, the velocity of the magnetic quasiparticles changes linearly with the applied current density. The final velocity *v*_*x*_ of the skyrmionium is larger than that of skyrmion and antiskyrmionite. This behavior can be verified by calculating the  term for each quasiparticle from eq 5. The skyrmionium has the most significant value of , resulting in the largest velocity *v*_*x*_ along the direction of the driving current. Figure 2c shows the Hall angle of movement for three magnetic quasiparticles with respect to the current densities, which is in good agreement with published theoretical calculations.^6^ Discrete data points represent micromagnetic simulation results, whereas the solid lines are results calculated from the Thiele equation. At smaller amplitude of current densities (<2 MA cm^–2^), micromagnetic simulation results of three magnetic quasiparticles fit well with the results calculated from the Thiele equation. However, at higher amplitude current densities (>2.5 MA cm^–2^), micromagnetic simulation results of the skyrmionium and antiskyrmionite start to derail from the theoretical predictions, whereas the skyrmion continues to follow the theoretical calculation of Thiele equation. This divergence can be explained by the shape distortion and rotation of the skyrmionium and antiskyrmionite under high current densities,^8,62^ which would be expected for the rigid particle approximation to show its limits for larger quasiparticles. As verification, we checked each quasiparticle’s topological charge and dissipation tensor with increasing current densities. The topological charge roughly remains the same for the three quasiparticles. In contrast, the dissipative tensor directly related to the skyrmion Hall angle in eq 4 varies a lot because of the distortion of quasiparticles under higher current densities. By increasing the current density from 0.3 MA cm^–2^ to 3.5 MA cm^–2^, the value of the  term in the dissipative tensor rises by 0.13, 14, and 20.6% for skyrmions, skyrmioniums, and antiskyrmionites, respectively, which explains well the divergence illustrated in Figure 2b, c. Note that when the applied current densities are large enough (>3.5 MA cm^–2^), we observe the annihilation of skyrmionium and antiskyrmionite at the edges of the nanotrack. Therefore, we mark the skyrmion Hall angles for the case of the skyrmionium and the antiskyrmionite with two red cross marks in Figure 2c under such high current densities.

Although multiple quasiparticles have been experimentally demonstrated in liquid crystals^7^ and bulk chiral magnets,^8,9^ it is vital to explore the conditions in which the skyrmion, skyrmionium, and antiskyrmionite may stably coexist in the same system. The coexistence of the three particles proposed in this work is outlined in the stability phase diagram, as shown in Figure 2d. The *x* axis and *y* axis represent the DMI and PMA constants, respectively. The color code illustrates the existence/nonexistence of each quasiparticle given a pair of DMI and PMA parameters. There are 31 × 26 = 806 data points displayed in Figure 2d, and every data point indicates whether any one of three quasiparticles can be stabilized under the corresponding parameters. For each set of the DMI and PMA constants, we configure the system with an initial ansatz that contains a single skyrmion, skyrmionium, and antiskyrmionite, respectively. We let the system equilibrate with the initial states and then mark every data point of Figure 2d with an existence/nonexistence for each quasiparticle according to whether the simulation results in the desired equilibrated state.

As shown in Figure 2d, the white-colored region marked with hollow circles represents the stabilization of the FM state; the yolk-colored region marked with filled circles is the skyrmion’s stabilization window; the olive region marked with triangles denotes the coexistence of the skyrmion and antiskyrmionite; the sky-blue area marked with rectangles represents the coexistence of skyrmion and skyrmionium; the navy-colored region marked with yellow stars is the target parameter window of this work that offers the coexistence of all three quasiparticles. Therefore, from Figure 2d, it can be summarized that the stabilization region of the skyrmion is the largest, whereas the skyrmionium and antiskyrmionite can stably exist in a subset parameter region of skyrmions. It should be noted that multiple magnetic parameters will contribute together in micromagnetic simulations, e.g., higher DMI constants are required when given high value of the exchange constants. The results shown in Figure 2d are in good agreements with reported simulation works.^4,11,35^ However, as for real devices in experiments, additive DMI can be achieved in tailored magnetic multilayer heterostructures.^63−65^ In previous studies reported in the literature,^4,11^ only specific types of quasiparticles, such as the skyrmion or the skyrmionium, have been proposed as information carriers in the device. However, in this work, we use multiple skyrmionic quasiparticles simultaneously in a single device, and this proposal is supported by the findings from Figure 2d that the three magnetic quasiparticles can coexist in a sufficiently wide parameter window for device usage.

### Thermal Stability of Magnetic Quasiparticles on the Track

**Figure 3:**
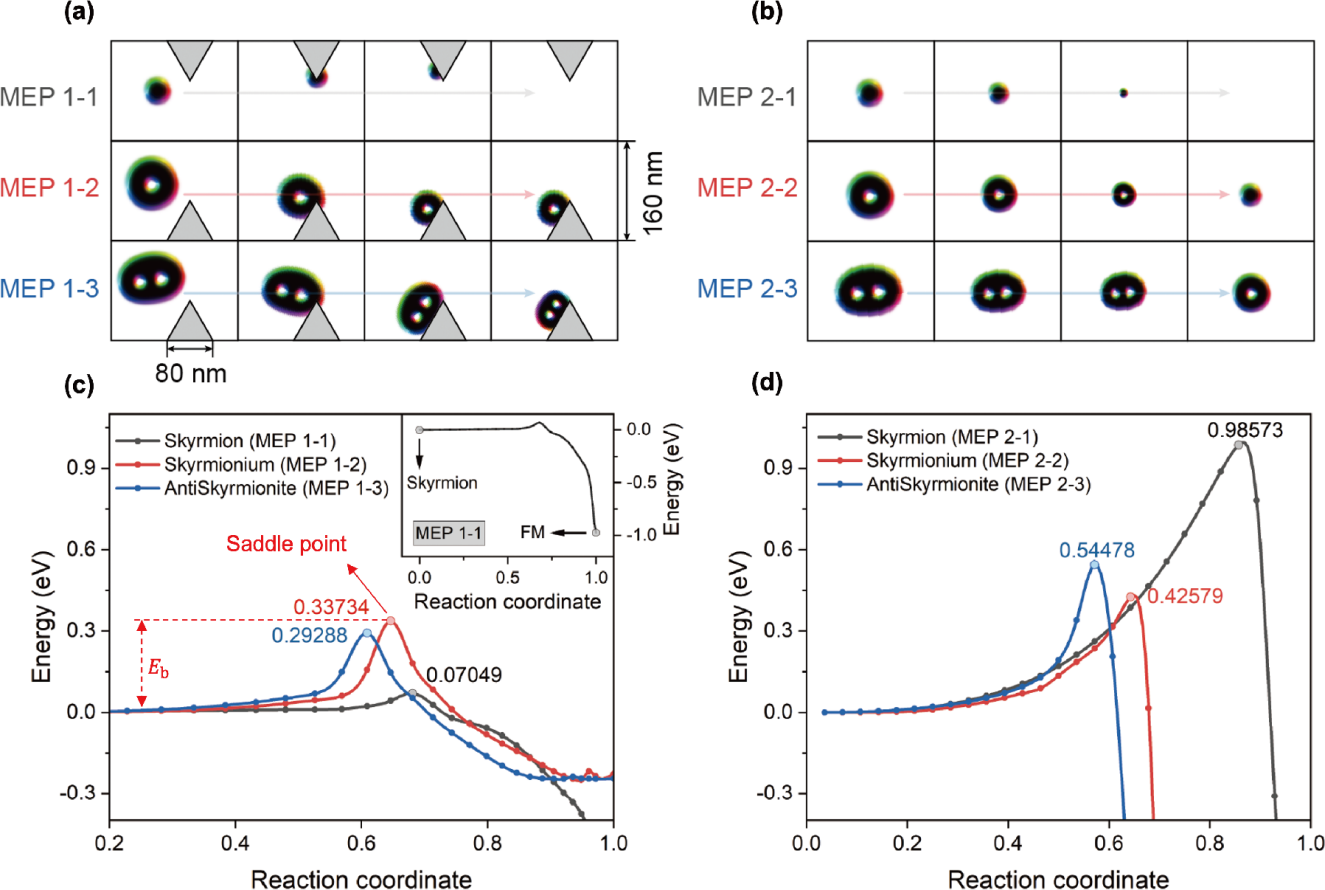
Minimum energy paths of a skyrmion, skyrmionium, and antiskyrmionite pinned and annihilated in the nanotrack. There are two MEPs for the annihilation: (a) magnetic quasiparticle gets pinned and annihilates at the vertex of the triangle notch, i.e., MEPs 1-1 to 1-3 and (b) a magnetic quasiparticle annihilates in the center of the nanotrack. Three paths are shown: skyrmion to the ferromagnetic state (MEP 2-1); skyrmionium to skyrmion (MEP 2-2); antiskyrmionite to skyrmionium (MEP 2-3). (c, d) Energy variation along with the MEPs illustrated in panels a and b, respectively. The inset of panel (c) shows the full MEP 1-1 with the larger range of the *y* axis illustrating the annihilation of a skyrmion state (energy 0 eV) toward the ferromagnetic state (energy −1 eV). The reaction coordinate is an order parameter representing the relative distance between the states in the configuration space, i.e., 0 stands for the initial state, and 1 represents the destination state. The energy barrier *E*_b_ of the transition is calculated by the difference of the total magnetic energy between a saddle point and an energy minimum. The results displayed in panel (d) are in perfect consistency with the calculations in our previous work.^34^ It should be noted that the energy for the quasiparticles shown in panels (c) and (d) is refined with respect to the quasiparticles’ corresponding initial states to facilitate better visualization and comparison.

**Figure 4:**
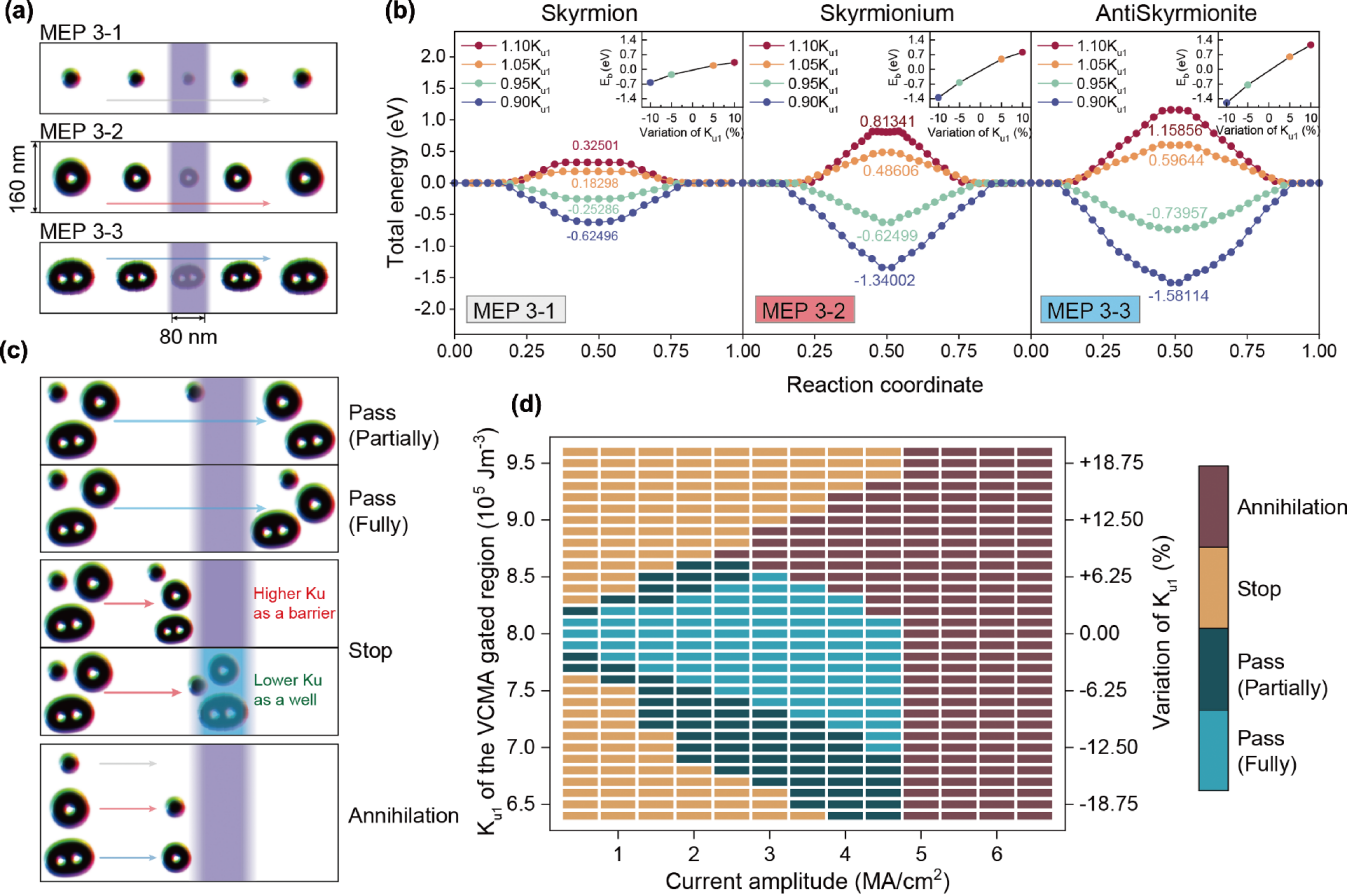
Phase diagram of the VCMA-gated region for skyrmion, skyrmionium, and antiskyrmionite. (a) Illustrations of MEPs of a skyrmion, skyrmionium, and antiskyrmionite passing through a VCMA region. (b) MEPs of the three magnetic quasiparticles as shown in panel (a) calculated using the NEBM. Insets illustrate the energy barrier *E*_b_ with varying *K*_u1_ of the VCMA-gated region. (c) Schematic illustration of the skyrmionic quasiparticles passing the VCMA-gated region in the nanotrack, where the purple shade represents the VCMA barrier and the sky-blue shade indicates a VCMA well. The quasiparticles propagate along the nanotrack when the constant driving current is applied and the VCMA gate is turned on. There exist four cases: (i) pass partially, (ii) pass entirely, (iii) stop, and (iv) annihilation of the quasiparticles. (d) Working window of the skyrmion, skyrmionium, and antiskyrmionite with different current densities and magnetic anisotropy constant *K*_u1_ was calculated with systematic micromagnetic simulations.

To further demonstrate the potential of the proposed VCMA-based interconnect and provide concrete results about the use of multiple quasiparticles simultaneously, we performed a thermal stability analysis with the NEBM,^66^ which has been widely used to calculate MEPs of multiple equilibrium states. When performing the NEBM, a transition between different local energy minimum states can be visualized as a path with respect to the reaction coordinate, defined by the cumulative sum of the distances between a sequence of configurations along the path. By defining the initial state and the destination state, the NEBM will determine the transition path with the minimum energy barrier, i.e., the MEP. It should be noted that the NEBM is usually used to calculate MEPs of multiple equilibrium states of the same system,^66^ and the NEBM is especially powerful in searching for MEPs among numerous possible ones. However, in this work, we calculated the energy barrier via the NEBM by constraining the target energy path (see Figure 3a,b, also in Figure 4a, which will be discussed in detail later) to plausible scenarios. We utilize the NEBM here to quantify the probabilities of different cases that elucidate the effect that the synchronization barriers (notches or VCMA gates) may play; for example, we compared the energy barriers of the quasiparticles’ annihilation in notch-based nanotracks and notch-free nanotracks, which can help decide the safer and stabler synchronization method for spintronic interconnect. More details of calculating the minimum energy paths (MEPs) and energy barriers can be found in Methods.

We first utilized the NEBM to estimate the possibility of pinning/annihilation of magnetic quasiparticles when there is a notch in the nanotrack, as proposed in recent numerical studies.^11,20,34^ Here, we compared two scenarios, shown in panels (a) and (b) in Figure 3, which represent the MEPs of a skyrmion, skyrmionium, and antiskyrmionite, pinned and annihilated by the triangle notch (MEPs 1-1 to 1-3) and collapsing in the center of a notch-free nanotrack (MEPs 2-1 to 2-3). We simulated a small section of the nanotrack of 160 nm width, and the notches in the simulations were realized by setting a nonmagnetic equilateral triangle region with a side length of 80 nm, as exhibited in Figure 3a. Skyrmionic quasiparticles may also annihilate at the nanotrack edges because of boundary roughness or Magnus force induced by large electrical current densities.^11,51^ With regards to edge annihilation of quasiparticles, as often reported in the literature, using high-*K* materials at the nanotrack edges can also prevent this from happening.^20,27,28^ Moreover, the latter concern has already been considered in section 2.2, e.g., by controlling the electrical current density below 4 MA cm^–2^ to prevent the magnetic quasiparticles from annihilating at the edge.

As for devices with a notch-based nanotrack, the annihilation of a magnetic quasiparticle takes place in two stages: it is first pinned by the notch; then it will annihilate on the site. However, our simulation results demonstrate that the skyrmion will immediately annihilate once it gets pinned by the notch, whereas the skyrmionium and antiskyrmionite show the two-stage annihilation behavior seen in Figure 3a. Although it is also possible for the skyrmionium and antiskyrmionite to be released/depinned from the notch, the pinning itself is sufficient to eliminate the information during the propagation process. Therefore, for the case of the notch-based nanotrack, we consider the energy barrier of magnetic quasiparticles being pinned at the vertex of the triangle notch. This probability can be quantified by determining the energy profile of the MEP illustrated in Figure 3a. The calculated energy barrier for the annihilation/pinning process of quasiparticles is shown in Figure 3c. Among the three quasiparticles, the skyrmion is most likely to annihilate at the notch with the energy barrier of 0.07 eV. Unlike the skyrmion that eventually degrades into the ferromagnetic ground state (see inset of Figure 3c), the skyrmionium and antiskyrmionite are pinned by the triangle notch afterward. Although the skyrmionium and antiskyrmionite exhibit a much more significant energy barrier of 0.34 and 0.29 eV, respectively, compared to the skyrmion, it is considerably smaller than the energy barrier for the collapsing process in the notch-free nanotrack shown in panels (b) and (d) in Figure 3. This means that under the same amount of current density, magnetic quasiparticles are much more likely to be pinned or annihilate in the notch-based nanotracks than in notch-free ones. Furthermore, we can quantify the stability by analyzing the results using the Arrhenius–Néel law to estimate the relaxation time^48,67^
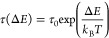
6where *f* = τ_0_^–1^ is the attempt frequency, *k*_B_ is the Boltzmann constant, and *T* is the temperature under consideration. Here, we assume *T* = 300 *K* as an estimation of quasiparticle lifetime at room temperature. The attempt frequency magnitude is difficult to obtain because the theory typically refers to macrospin systems. In the literature, a 1 × 10^9^ to 1 × 10^12^ Hz frequency is typically used.^48,67,68^ However, there is a debate about the value of the attempt frequency,^69^ as it can be as large as 1 × 10^21^ Hz. Precisely calculating the lifetime of quasiparticles needs a dedicated investigation and is beyond the scope of this paper. We choose a typical value of 1 × 10^12^ Hz for the attempt frequency here and give an approximate estimation and comparison of the quasiparticle lifetimes. As for MEPs 1-1, 1-2, 1-3, 2-1, 2-2, and 2-3 shown in Figure 3, we calculate the lifetime of each one using eq 6 as 0.015, 458.6, 83.6, 3.66 × 10^13^, 1.43 × 10^4^, and 1.4 × 10^6^ ns, respectively. The results suggest that the magnetic quasiparticles carry a relatively shorter lifetime due to the likely pinning and annihilation at the notches. Therefore, the notch-based nanotrack would be fragile if we employ it in realistic devices, let alone under room temperature thermal fluctuations. In comparison, quasiparticles show a tremendously improved stability in notch-free nanotracks. As shown in Figure 3b,d, quasiparticles exhibit a much longer lifetime in the nanotrack, especially for magnetic skyrmions, whose lifetime skyrockets from 0.015 ns in notch-based nanotrack to 3.66 × 10^13^ ns in the notch-free nanotrack. The notches induce an impressive degradation in the device stability by 15 orders of magnitude. Note that the lifetime estimated from energy barriers indicates the thermal stability and annihilation possibility of quasiparticles rather than the precise operation times for realistic devices. In other words, a larger energy barrier contributes to a longer lifetime, which results in better thermal stability of quasiparticles. Therefore, the target of the device design is to obtain larger energy barriers of quasiparticles to withstand greater external disturbances, e.g., thermal fluctuations and electric currents. As a result, quasiparticles will exhibit faster and more reliable operations in the device. In the following, we explore whether VCMA-gated synchronizers in the device provide better thermal stability for magnetic quasiparticles than notches.

As for the notch-free, VCMA-based interconnect device proposed in this work (see Figure 1), we can roughly estimate the thermal stability of magnetic quasiparticles according to the collapse process illustrated in Figure 3b. In this case, the magnetic quasiparticles are most likely to annihilate within VCMA-gated regions or at the corner of the VCMA-gated region and nanotrack edges. The probability of this procedure can be qualitatively described by the stability of quasiparticles against switching into others (i.e., skyrmionium to skyrmion, antiskyrmionite to skyrmionium, shown in Figure 3b), where all three quasiparticles show larger energy barriers in notch-free devices than notch-based devices. In addition, compared to the strategically etched notches, the VCMA-gated synchronizers proposed in this work have several advantages. First, the VCMA-based interconnect shows better scalability than the notch-based one. Indeed, a single VCMA gate can be used to synchronize multiple quasiparticles, whereas in the notch-based interconnect device, each magnetic quasiparticle requires one notch artificially etched in a specific position.^34^ Second, the VCMA-controlled gate is fully tunable through voltage and can, therefore, remedy process variations during fabrication. In contrast, notch-based devices are more sensitive to fabrication process variations and, more importantly, are adversely affected by these variations. Therefore, comparison of the energy barrier calculations in Figure 3c,d and the reasoning above suggest that magnetic quasiparticles should exhibit better thermal stability and lower probability of annihilation in VCMA-based interconnects than notch-based ones. In the following, we will also investigate the role of VCMA gates.

### Pipelined Spintronic Interconnect Synchronized by VCMA Gates

By switching VCMA gates on and off in the nanotrack, we effectively manage the lateral energy distribution of the system, which will result in the stop and pass of the information carriers. To obtain the energy distribution of the nanotrack when turning on the VCMA gates, we calculated the energy barrier via the NEBM for each quasiparticle when passing a VCMA-gated region where the magnetic anisotropy constant *K*_u1_ varies. As shown in Figure 4a, the width of the nanotrack under simulation is 160 nm and the VCMA-gated region is 80 nm. Energy profiles of the skyrmion, skyrmionium, and antiskyrmionite moving along the track (i.e., MEPs 3-1, 3-2, and 3-3, respectively) were calculated in Figure 4b, where the *K*_u1_ values of VCMA-gated regions were changed from 0.90*K*_u1_ to 1.10*K*_u1_. The insets in Figure 4b describe the near-linear relation between the energy barrier *E*_b_ for quasiparticles crossing the VCMA-gated region and the variation in *K*_u1_ of the VCMA region. Note that the VCMA range can be as large as ±40% according to the literature,^44^ but a relatively smaller and feasible range of ±10% is considered in this work.

As for each magnetic quasiparticle, a higher *K*_u1_ variation (Δ*K*_u1_) of the VCMA-gated region leads to more significant energy differences in and out of the VCMA-gated region. Comparing the same amount of Δ*K*_u1_ with a positive and negative sign, the negative one leads to a higher energy barrier. The difference in energy barriers of ±Δ*K*_u1_ could be attributed to the shape shrinking/expanding of quasiparticles when the PMA constant is adjusted, in addition to the energy change purely due to the PMA. As a result, the calculated energy barrier for the +Δ*K*_u1_ VCMA gate is reduced, while the energy barrier for the −Δ*K*_u1_ VCMA gate is strengthened. Such asymmetry of energy barriers may result in similar asymmetric effects on device functionality where the VCMA gates are deployed. These results are in good agreement with other simulations on the pinning/depinning properties of individual skyrmions via VCMA gates.^23,70^ Therefore, the VCMA-gated regions with lower *K*_u1_ could potentially serve as registers to store state information on quasiparticles. Among these three quasiparticles, the skyrmion experiences the lowest energy barrier passing the VCMA gate than the skyrmionium and the antiskyrmionite under the same Δ*K*_u1_. For example, if we change PMA to be positive 10% by applying a voltage on the VCMA gate, the energy barrier for skyrmion, skyrmionium and antiskyrmionite crossing the region is calculated as 0.325, 0.813, and 1.158 eV, respectively. However, this does not mean that skyrmions are more likely to cross over the VCMA-gated region than skyrmioniums and antiskyrmionites under the same amplitude of current density, because the additional energy due to the current-induced spin-transfer torques is different for each quasiparticle. In fact, from the micromagnetic simulations, we noted that by increasing the current density in the nanotrack, the antiskyrmionite is the first to cross the VCMA-controlled barrier while the other two are stuck.

As discussed above, we can achieve either a higher or lower PMA and energy barrier by applying a positive or negative voltage to the VCMA gates. As shown in Figure 4c, the region with higher PMA serves as an energy barrier to stop the magnetic textures (for some transition values, one or two of the particles would pass the VCMA-gated region, which we refer to as a partial pass here) and the region with lower PMA offers a potential well to trap the quasiparticles. From the results of Figure 4b, we observe that if the applied current density is smaller than the critical value that compensates the energy barrier/potential well, quasiparticles would be stopped by the VCMA gate. Otherwise, the quasiparticles will pass the VCMA-gated region (see top panel in Figure 4c). Furthermore, if the energy of the applied current is larger than the energy barriers shown in Figure 3b,d, the quasiparticles will annihilate during propagation.

There are four possible cases: (i) pass partially, (ii) pass fully, (iii) stop, and (iv) annihilation, depicted in Figure 4c. We then performed a series of simulations of a nanotrack with a VCMA gate placed in the middle of the track. By scanning the *K*_u1_ value of the VCMA-gated region and the current amplitude injected in the nanotrack, we obtained the working window for the pass/stop/annihilation status of quasiparticles in the device, as shown in Figure 4d. We considered the simulation containing all three quasiparticles rather than having each one individually. Because in real devices, we use three of them together, and we need to guide the device design with the strictest condition where all three particles need to cross or be stopped by the VCMA gate. The results organized in Figure 4d fit well with our prediction in the discussion above. Under smaller current densities, the VCMA gate with high Δ*K*_u1_ will stop the quasiparticles, whereas quasiparticles will pass the VCMA gate with lower Δ*K*_u1_. Above high current densities (>4.5 MA cm^–2^), the annihilation of quasiparticles will be seen. Another significant result in Figure 4d is the asymmetry of the “annihilation” phase along with *K*_u1_, which could be explained by the asymmetry of the energy barrier *E*_b_ of each quasiparticle with K_u1_ in Figure 4b. For negative Δ*K*_u1_, the quasiparticles find it difficult to escape from the VCMA well due to the higher *E*_b_. So, in the bottom half of Figure 4d, the quasiparticles will not annihilate until the current density increases to larger than 4.5 MA cm^–2^, which is the threshold value to prevent quasiparticles from collapsing.

**Figure 5:**
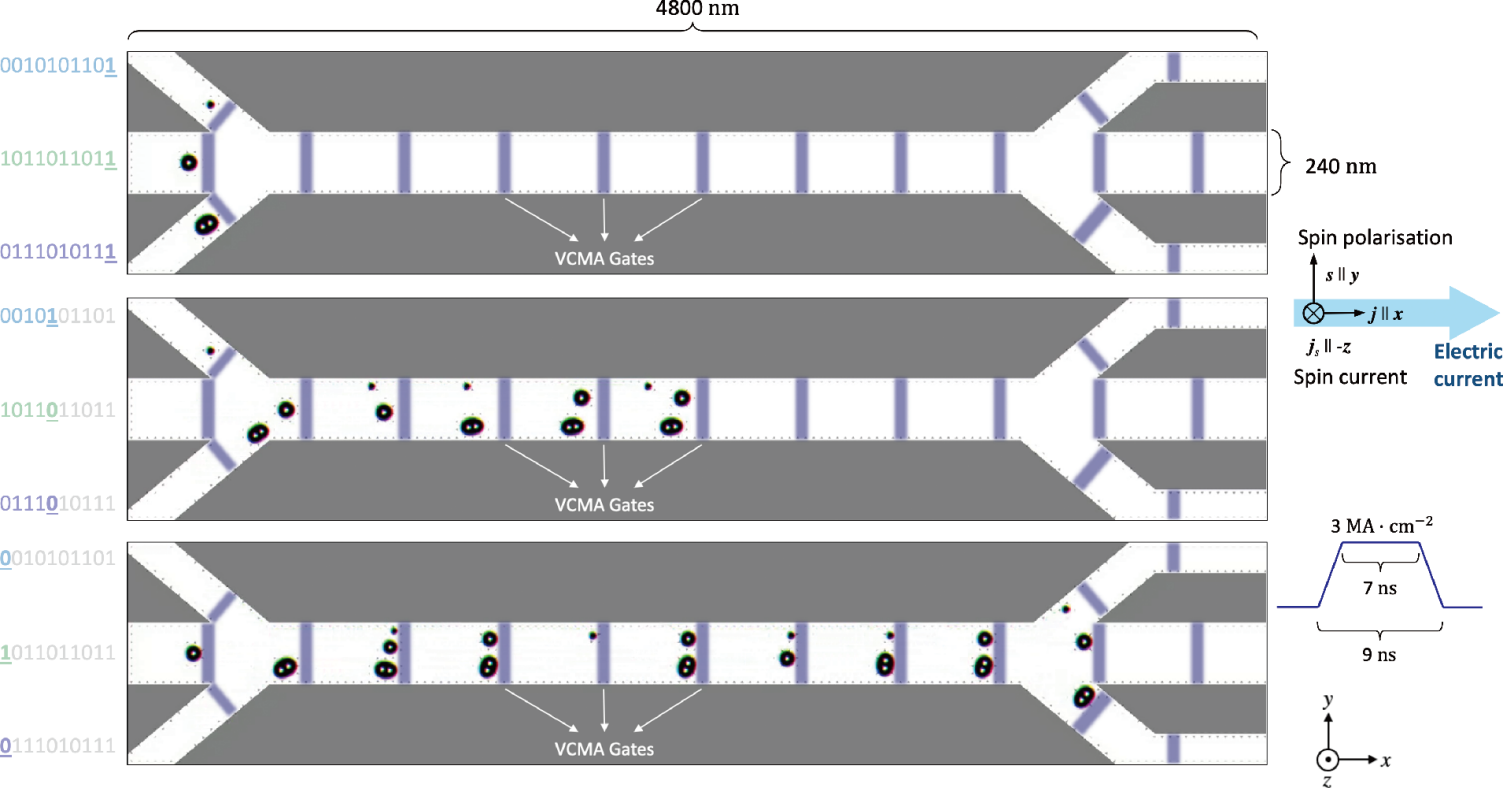
Micromagnetic simulations of the pipelined spintronic interconnect. The racetrack is divided into separate regions via VCMA gates. The device is powered by a series of electrical pulses perpendicular to the plane (CPP) with an amplitude of 3 MA cm^–2^ for 9 ns. The pulse consists of 1 ns rising edge, 7 ns constant current, and 1 ns falling edge. The width of the main racetrack is 240 nm and the length of the device 4800 nm, and the spacing between adjacent VCMA gates is 400 nm.

On the basis of the results discussed above, we carefully designed a scheme for a pipelined spintronic interconnect, as shown in Figure 5. The length of the nanotrack is 4800 nm, and the width is 240 nm. As schematically illustrated in Figure 1, the device has a three-branch encoder and a three-branch decoder to encode and decode three sequences of information signals. In the simulation, we placed eight VCMA gates on the main track and several VCMA gates near the nucleation and detection heads in the branches. As for the setup of the VCMA effect, we set a 5% variance of *K*_u1_ for VCMA gates during the simulation, which required a voltage less than 1 V according to eq 2. It should be noted that the number of VCMA gates employed in the device is determined by the number of bits. Here, we want to examine the transmission of one byte (eight bits) information, which is usually the smallest addressable unit of memory in many computer architectures. Moreover, because of the nonvolatility of skyrmionic quasiparticles, such a design can also be used to implement an 8-bit shift register.

The presence of the magnetic quasiparticles encodes logic “1″, while the absence of the quasiparticles corresponds to logic “0”. A series of driving current pulses is periodically applied in the HM layer in the direction of the *x* axis with a 1 ns rising edge and a 1 ns falling edge. The spin current is therefore injected perpendicular to the FM plane (in the direction of −*z*) with the spin polarization in the +*y* direction. We include rising and falling edges in our pulses because we want to provide realistic operating conditions of current injection for experiments/devices. At the same time, the rising edge of current pulses can provide the device with an initializing process, and the falling edge can ensure all the quasiparticles reach and are stopped by the VCMA-gated synchronizer simultaneously to protect the order of information sequences. It should be noted that in our simulation of the pipelined interconnect device, we obtain and stabilize the magnetic quasiparticles by setting up initial ansatzes with their shapes and letting them equilibrate in situ in the system. In the simulation shown in Figure 5, we send and fully transmit three sequences of information signals “1011010100 (blue), 1101101101 (green), 1110101110 (purple)” serially. In Figure 5, the information sequences are printed in reverse order. Information bits are multiplexed, transmitted through the pipeline, and demultiplexed simultaneously, tripling the bandwidth of an interconnect that carries only a single information carrier (i.e., a magnetic particle). Therefore, with the help of VCMA gates, the proposed skyrmionic interconnect inherently supports data pipelining, where the interconnect throughput is boosted, despite of a 10-pulse long latency for the first piece of data to be received. Note that such latency is given in the context of the maximum interconnect throughput, where the electric pulses are sent after previous one immediately without rest time. These results illustrate potential applications of our proposed spintronic interconnect device.

## Discussion

### Tunable Interconnect Performance and Analysis on the Energy Efficiency

We have demonstrated the whole device operation flow, and the pipelined scheme of the voltage-controlled spintronic interconnect device. To evaluate the possible benefits of our proposed interconnect for future integrated systems, we describe the tunability of the device performance and compare its energy efficiency with copper interconnects, which are commonly used in the mainstream CMOS technology.

**Figure 6:**
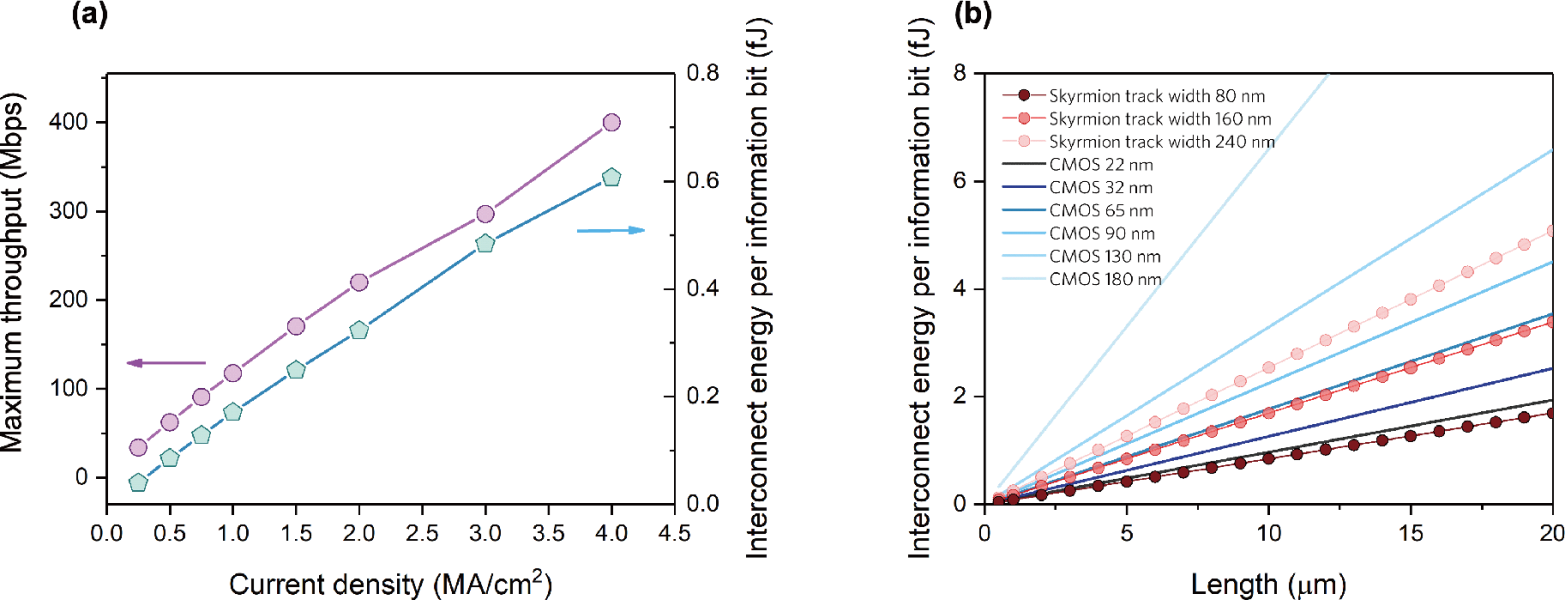
Tunability of the device performance and energy consumption. (a) Maximum throughput and energy consumed for single information bit transmission of the proposed pipelined device under different current densities. (b) Comparison of the energy efficiency of the proposed pipeline spintronic interconnects with the copper interconnects in CMOS technology nodes varying from 180 to 22 nm in the fabrication process.

The performance of interconnects can be quantified by their maximum throughput as well as their energy efficiency. The throughput of the pipelined interconnect introduced in Figure 5 is given by^71^
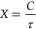
7where *X* is the maximum throughput of the proposed interconnect device and *C* is the total amount of transferred data bits within the time τ. As indicated from Figure 6a, the device throughput can be effectively tuned by adjusting the current densities. Because the skyrmionium and antiskyrmionite annihilate at *J*_e_ > 4 MA cm^–2^, the current densities here are limited to 4 MA cm^–2^, where τ = 7.5 ns is obtained from the simulations to propagate an information package between adjacent VCMA gates. This sets an upper limit of 400 Mbps maximum throughput of the device (calculated via eq 7 with *C* = 3 bits), shown in Figure 6a. Regarding the interconnect latency, there is a 10-pulse long latency between the two ends (i.e., sender and the receiver), i.e., 90 ns calculated by the electrical pulse utilized in the pipelined scheme of Figure 5. At the same time, this latency can also be tuned by changing the current densities. For example, if we want to achieve a 10-time higher interconnect throughput we increase the current density of the applied pulses such that the quasiparticles propagate faster, and the required pulse width is shortened by 10 times. Therefore, the latency will be correspondingly reduced by 10 times. As shown in Figure 6a, there is an almost linear relationship between the maximum device throughput and current densities, which indicates the effective tunability of the device performance merely by modifying the current supply.

The energy consumption for information transmission of the proposed spintronic interconnect is given by
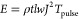
8where *E* is the energy consumption, ρ is the resistivity of the HM layer, *t* is the thickness of the HM layer, *l* is the length of the racetrack, *w* is the width of the nanotrack, *J* is the density of the charge current applied in the HM layer, and *T*_pulse_ is the pulse duration for the proposed pipelined spintronic interconnect. The values of the parameters are chosen as follows: *l* = 400 nm (distance between two VCMA gates); *t* = 3.8 nm (the spin diffusion length of Pt^50,72^); *w* = 240 nm; ρ = 50 μΩ cm for 3.8 nm Pt thin film.^50^ We integrate *J*^2^*T*_pulse_ with the current pulse shape depicted in Figure 5. The transmission energy per information bit with respect to current density is also shown in Figure 6a. Like the linear tunability of the interconnect maximum throughput, the transmission energy rises linearly with respect to the applied current as well. The results in Figure 6a provide design guidelines for future implementations of such interconnect devices, which can tune the device performance within the power limitations in practical devices and application scenarios.

Finally, we evaluate the spintronic interconnect by comparing it with conventional CMOS technology to offer better insight into the suitability of the skyrmionic interconnect devices. We calculate the energy consumption of the copper interconnects across different CMOS technology nodes. In CMOS interconnects, the energy consumption to transfer a bit via a copper interconnect is described by
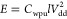
9where *C*_wpu_ is the wire capacitance per unit length, *l* is the length of the copper wire, and *V*_dd_ is the voltage supply. For a fair comparison, we choose length *l* = 400 nm, the same with the spintronic interconnect. Copper wire capacitance *C*_wpu_ and the voltage supply *V*_dd_ of different CMOS technologies are estimated via the predictive technology model (PTM).^73^ The detailed parameters of copper interconnect and calculation via PTM can be found in Methods. Figure 6b compares the transmission energy with respect to interconnect length for the proposed spintronic interconnect device and the copper interconnect in 180 nm down to 22 nm CMOS technology nodes. As for the spintronic interconnect, we choose the transmission energy for a 100 Mbps throughput from Figure 6a. As shown in Figure 6b, the estimated energy efficiency of the spintronic interconnect with a 240 nm width nanotrack is better than the copper interconnect in the 130 nm CMOS technology node and comparable with (slightly worse than) a copper interconnect in the 90 nm CMOS technology node. By narrowing the width of the skyrmion nanotrack, the transmission energy can be further reduced. We have also calculated the transmission energy of the spintronic interconnect devices whose widths are 160 and 80 nm, respectively, by assuming that the antiskyrmionite would be stabilized at the 80 nm width nanotrack. The 160 nm width spintronic interconnect exhibits a similar energy efficiency with copper interconnect in 65 nm CMOS node, whereas the 80 nm width spintronic interconnect presents comparable energy efficiency with that of the 22 nm CMOS node. According to eq 8, reducing the width of the nanotrack *w* can directly decrease the data transmission energy since the calculated energy *E* is linearly dependent on *w*. However, the width of the nanotrack cannot be narrower than the lateral dimensions of magnetic quasiparticles, which varies in different material systems. In this work, the diameters of the skyrmion, skyrmionium, and antiskyrmionite (minor diameter of its ellipse shape) are estimated to be approximately 30, 60, and 80 nm. Consequently, we set 80 nm as the lower limit of the nanotrack width *w*.

The information transmission energy of the spintronic interconnect device determined here also includes the multiplexing and demultiplexing procedures, while the calculated energy for copper interconnects purely accounts for bit transmission without considering any control signal. At the same time in spintronic interconnects additional energy is spent for switching on/off the VCMA gates that guide the quasiparticles along the nanotrack, which can be implemented by periphery circuits. For simplicity, we assume the energy to switch VCMA gates in spintronic interconnect is comparable to the energy to send control signals in CMOS interconnects. Therefore, we use the eqs 8 and 9 to directly compare data propagation energy between spintronic and copper interconnect in CMOS, as shown in Figure 6b. The concept of spintronic interconnects with multiple quasiparticles is even more advantageous since data transmission is accompanied by automatic multiplexing and demultiplexing at both ends. The nonvolatility and bidirectionality of the devices are also noteworthy benefits. We anticipate that future work on spintronic interconnect devices could include: (i) evaluating spintronic interconnect devices in realistic conditions, e.g., magnetic multilayer structures with realistic material grains and defects at room temperature, (ii) research on how to enhance the thermal stability of quasiparticles in the device, (iii) the detailed nucleation process of magnetic quasiparticles, especially when thermal fluctuations and stochastic behaviors are considered, (iv) investigating strategies to expand throughput for given energy efficiency, and (v) experimental realizations of the proposed interconnect device.

## Conclusions

In this work, we proposed to utilize multiple magnetic quasiparticles as information carriers (i.e., magnetic skyrmion, skyrmionium, and antiskyrmionite) in a spintronic interconnect device based on voltage-controlled magnetic anisotropy (VCMA) gates. The device can achieve automatic multiplexing/demultiplexing and simultaneous transmission of multiple information signals. Combining theoretical analysis with micromagnetic simulations, we demonstrated the distinct current-driven behavior of different magnetic quasiparticles. Through the NEBM, we showed that quasiparticles in VCMA-based interconnect are more thermodynamically stable than notch-based structures. A pipelined interconnect was then illustrated by embedding VCMA-based gates as synchronizers. Lastly, we showed that the energy efficiency of the skyrmionic interconnect is comparable to copper interconnects in CMOS technologies. This work significantly widens the possibilities for all-magnetic spintronic devices with multiple quasiparticles as information carriers and should be relevant in a future potential holistic spintronic nanocomputing paradigm.

## Methods

### Micromagnetic Simulation

The micromagnetic simulations were performed using the GPU-accelerated micromagnetic package MuMax3.^47^ The time-dependent magnetization dynamics are conducted by the Landau–Lifshitz–Gilbert (LLG) equation with an additional term accounting for the Slonczewski spin–orbit torque:^74^
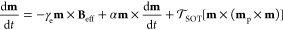
10where **m** = **M**/*M*_s_ is the reduced magnetization, *M*_s_ is the saturation magnetization, γ_e_ = 1.76 × 10^11^ T^–1^ s^–1^ is the gyromagnetic ratio of an electron, **B**_eff_ is the space- and time-dependent effective magnetic field, α is the dimensionless Gilbert damping parameter,  is the SOT efficiency, where ℏ is the reduced Planck constant, *J*_e_ is the electron current density, θ_SH_ is the spin Hall angle, *e* is the electron charge, *t* is the thickness of FM layer, and **m**_p_ is the polarization direction of the spin current due to the spin Hall effect. The micromagnetic energy density ε(**m**) is a function of **m**, which contains the Heisenberg exchange energy term, the anisotropy energy term, the Zeeman energy term, the magnetostatic energy term and the DMI energy term. The material parameters to perform the simulations are chosen following refs (4),^11^, and (21) damping parameter α = 0.3, interfacial DMI constant *D*_int_ = 3.5 mJ m^–2^, saturation magnetization *M*_s_ = 580 kA m^–1^, the spin Hall polarization θ_SH_ = 0.6 to enhance the spin Hall effect, the uniaxial out-of-plane magnetic anisotropy *K*_u1_ = 800 kJ m^–3^, the polarization of the spin current is in the +*y* direction, and the exchange constant is assumed to be *A* = 15 pJ m^–1^. To ensure the accuracy of calculation, the mesh size of discretization is set to 1 × 1 × 1 nm^3^, which is much smaller than the exchange length  and DMI length *l*_DMI_ = 2*A*/*D*_int_ = 8.57 nm. The thickness of the heavy metal layer is 3.8 nm, which is chosen as the spin diffusion length of Pt.^50^ An external magnetic field of 10 mT in the out-of-plane direction is applied. The edges of 5 nm thickness with higher magnetic anisotropy *K*_u1,high_ = 900 kJ m^–3^ is set for the device to avoid magnetic quasiparticle annihilation at the nanotrack edges.

### Solution to the Thiele Equation in the Presence of the SOT

As introduced in the main text, there are two situations to be considered: (1) propagation of the magnetic quasiparticle far from the edge; and (2) the quasiparticle eventually moving along the direction of the applied current along the nanotrack edge. By calculating the dissipative tensor  and the driving torque tensor  via MATLAB using the micromagnetic profiles of the skyrmion, skyrmionium, and antiskyrmionite, the tensor  and  have the following shapes,
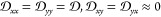
11
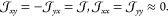
12Here we assume that the magnetic quasiparticle does not perform displacement in *z* direction. For a magnetic quasiparticle propagating in a nanotrack with periodical boundary conditions along *x* (*U*(**r**) = *U*(*y*)), the Thiele equation in eq 3 reads
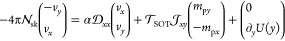
13For the magnetic quasiparticles far from the edge, ∂_*y*_*U*(*y*) = 0. Assuming the injected spins along the −*y* direction, i.e., *m*_p*x*_ = 0, *m*_p*y*_ = −1, eq 13 can be further simplified to,
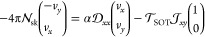
14Solving eq 14, we can extract the Hall angle of the particle movement as,
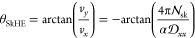
15For the magnetic quasiparticles steadily moving along the track edge (*v*_*y*_ = 0), eq 13 can be written as,
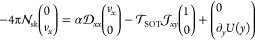
16We obtain the velocity of the magnetic quasiparticle in *x* direction
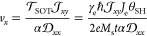
17The results from the micromagnetic simulations and the Thiele equation are summarized in Table 1 below.

**Table 1:** Results of the Simulations and Theoretical Predictions with Thiele Equation

magnetic quasiparticle	*N*_sk_	*N*_sk_ MATLAB	Thiele	Thiele (nm)	θ_SkHE_ Sim. (deg)	θ_SkHE_ Thiele (deg)	*v_x_* Sim. (m/s)
skyrmion	–1	–0.9998	22.58	148.8	61.5	61.66	49.7
skyrmionium	0	–0.0002	64.89	429.8	–0.2	0	55.0
antiskyrmionite	1	0.9995	102.76	676.26	–26.3	–24.88	51.4

### Predictive Technology Model

The PTM can provide accurate, customizable, and predictive model files for transistor and interconnect technologies.^73^ It is compatible with standard circuit simulators (e.g., SPICE) and scalable with disparate process variations. PTM is broadly used for pathfinding activities before a semiconductor technology is fully developed. Therefore, it is an ideal tool to help us calculate the information transmission energy for CMOS technologies. To calculate the transmission energy via eq 9, we need the value of parameters *C*_wpu_ and *V*_dd_. The voltage supply *V*_dd_ is taken from ref (75), and the dimensions are estimated from the technology sheets in ref (76). The wire capacitance per unit length of the copper interconnect *C*_wpu_ can be estimated by using the PTM with salient parameters of the copper interconnect across several generations of CMOS technology including, for instance, width, space, thickness, height, and dielectric. The typical values of the above parameters for different generations of CMOS technology are summarized in Table 2. The calculated *C*_wpu_ and transmission energy per information bit are also listed in Table 2.

**Table 2:** Parameters of the Copper Interconnect in Various Generations of CMOS Technology Utilized in the PTM and the Calculated Wire Capacitance and Transmission Energy Per Information Bit

CMOS tech.	*V*_dd_ (V)	width (nm)	space (nm)	thickness (nm)	height (nm)	dielectric κ	*C*_wpu_ (fF/mm)	energy (fJ) length 400 nm
22 nm	0.9	35	35	70	70	1.8	119.62	0.04845
32 nm	1.0	50	50	100	100	1.9	126.22	0.06311
65 nm	1.1	100	100	200	200	2.2	146.20	0.08845
90 nm	1.1	150	150	300	300	2.8	186.08	0.11258
130 nm	1.2	200	200	450	450	3.2	228.66	0.16464
180 nm	1.8	280	280	650	650	3.5	255.32	0.33009

### Minimum Energy Path Calculations

The NEBM^66^ has been widely used to calculate the minimum energy paths (MEPs) of multiple equilibrium states (energy minima). A transition between different states can be visualized as a path with respect to the reaction coordinate, defined by the cumulative sum of the distances between a sequence of configurations along the path. In the magnetic system, a minimum energy path refers to the path that requires the minimal cost of total energy, and an energy barrier *E*_b_ of the transition is calculated by the difference between an energy maximum (saddle point) and an energy minimum. Different from the Monte Carlo method, which samples the most probable transition paths, the NEBM starts from an initial guess of a path, and the algorithm minimizes the path by lowering the saddle point, by analogy with tensioning an elastic band across a mountain. In this work, micromagnetic simulations of this part are performed using Fidimag^48^ for calculations of equilibrium states of topological spin textures, where magnetizations are relaxed based on the Landau–Lifshitz equation of motion (eq 10), followed by the minimization of the total energy by the steepest descent method.^77^ Magnetic parameters utilized in Fidimag are the same as those in MuMax3. The NEBM method is then used to calculate MEPs between the proposed equilibrium states to quantify the energy barriers along with the transitions.
