# Correction to “Voltage-Controlled Skyrmionic Interconnect with Multiple Magnetic Information Carriers”

**DOI:** 10.1021/acsami.3c02434

**Published:** 2023-03-20

**Authors:** Runze Chen, Yu Li, Vasilis F. Pavlidis, Christoforos Moutafis

**Affiliations:** † Nano Engineering and Spintronic Technologies (NEST) research group, Department of Computer Science, The University of Manchester, Manchester M13 9PL, United Kingdom; ‡ Advanced Processor Technologies (APT) research group, Department of Computer Science, The University of Manchester, Manchester M13 9PL, United Kingdom

The University of Manchester conducted an investigation under the Code of Practice for Investigating Concerns about the Conduct of Research in relation to the authorship dispute of this paper. The Panel of Investigation recommended inclusion of Christoforos Moutafis and Vasilis Pavlidis as authors and a minor correction to the paper with regard to the author contributions, and all parties are in agreement with this recommendation.

Therefore, this correction amends the authorship, author contributions, and the acknowledgments in order to correct the academic record and maintain academic ethics.

